# Health effects of air pollution on length of respiratory cancer survival

**DOI:** 10.1186/1471-2458-13-800

**Published:** 2013-09-03

**Authors:** Xiaohui Xu, Sandie Ha, Haidong Kan, Hui Hu, Barbara A Curbow, Claudia TK Lissaker

**Affiliations:** 1 Department of Epidemiology, College of Public Health and Health Professions and College of Medicine, University of Florida, PO Box 100231, Gainesville, FL 32610, USA; 2 Department of Environmental Health, School of Public Health, Fudan University, Shanghai, China; 3 Department of Behavioral Science and Community Health, College of Public Health and Health Professions, University of Florida, Gainesville, FL 32610, USA

**Keywords:** Air pollution, Cancer, Cancer survival, Respiratory cancer, Lung cancer

## Abstract

**Background:**

Air pollution has been extensively and consistently linked with mortality. However, no study has investigated the health effects of air pollution on length of survival among diagnosed respiratory cancer patients.

**Methods:**

In this study, we conducted a population-based study to investigate if air pollution exposure has adverse effects on survival time of respiratory cancer cases in Los Angeles (LA), CA and Honolulu, HI. We selected all White respiratory cancer patients in the two study areas from the 1992–2008 Surveillance Epidemiology and End Results cancer data. Death from respiratory cancer and length of survival were the main outcomes.

**Results:**

Kaplan-Meier survival analysis shows that all respiratory cancer cases exposed to high air pollution referring to the individuals from LA had a significantly shorter survival time than the low pollution exposure group referring to those from Honolulu without adjusting for other covariates (p <0.0001). Moreover, the results from the Cox Proportional-Hazards models suggest that exposure to particles less than 10 micrometers in diameter (PM_10_) was associated with an increased risk of cancer death (HR = 1.48, 95% CI: 1.44-1.52 per 10 μg/m^3^ increase in PM_10_) after adjusting for demographic factors and cancer characteristics. Similar results were observed for particles less than 2.5 micrometers in diameter and ozone.

**Conclusion:**

Our study indicates that air pollution may have deleterious effects on the length of survival among White respiratory cancer patients. This study calls for attention to preventive effort from air pollution for this susceptible population in standard cancer patient care. The findings from this study warrant further investigation.

## Background

Cancer is the second leading cause of death in the US, accounting for over 500,000 deaths annually [1]. Worldwide, over 7.6 million people died from cancer in 2008 based on the GLOBOCAN estimates [2]. Respiratory cancer, especially lung cancer, is one of the most commonly diagnosed cancers as well as the leading cause of cancer death. In the United States alone, over 200,000 new lung cancer cases were diagnosed, and of whom nearly 161,250 died in 2011 [3]. Reducing the death rate from respiratory cancer is still a challenging mission although a slight progress has been made over the past years.

Epidemiological studies have shown consistent evidence of short-term health effects of air pollution on cardiopulmonary morbidity and mortality using time-series analyses and case-crossover designs [4-7]. Moreover, air pollution as a risk factor of respiratory cancer has been reported in several studies [8-12]. However, previous studies revealed that major effects of air pollution have been observed among the susceptible groups such as children, elderly and persons with chronic conditions including diabetes and heart disease [13,14]. Therefore, for research on air pollution and respiratory cancer, it may be also interesting to investigate the effects of air pollution on the length of survival among respiratory cancer patients who are already severely stressed.

To our knowledge, no studies have been conducted to examine the long-term effects of air pollution on any type of cancer survival. Inhalation of polluted air may have significant impacts on survival among respiratory cancer cases. As industrialization and urbanization continues to advance, global increase in industrial processes and energy consumption results in burning a large amount of fossil fuel including gasoline, coal and natural gas. These activities are continuing to release toxic pollutants into the air we breathe [15]. Despite the significant effort to achieve cleaner air following the Clean Air Act in 1970 in the USA [16], air pollution remains a significant public health problem [17]. According to the State of the Air 2012 report by the American Lung Association, about four out of ten people are living in counties that receive an F for air quality in the United States [18]. Given the high prevalence of both air pollution and respiratory cancer, it is urgent and critical to understand if exposure to air pollution has adverse effects on respiratory cancer survival.

The purpose of this population-based study was to investigate adverse effects of air pollution on respiratory cancer survival using the 1992–2008 Surveillance Epidemiology and End Results (SEER) cancer data in the U.S. In addition, this study further investigated whether air pollution has impacts on survival of cancer at a specific site of respiratory system.

## Methods

### Study locations

In this study, we selected Honolulu, HI and Los Angeles (LA), CA as two study areas because these two locations provided different air pollution exposure levels. Honolulu is one of the cleanest areas while LA is on the list of most polluted cities in U.S. [18]. We analyzed ambient air pollution levels in these two study areas using the U.S Environmental Protection Agency monitored air data. The means of annual averages of concentrations of criteria air pollutants including particles less than 10 micrometers in diameter (PM_10_), particles less than 2.5 micrometers in diameter (PM_2.5_) and Ozone (O_3_) between 1992–2008 in LA, CA and Honolulu, HI are listed in Additional file 1: Table S1. The trends of annual concentrations of PM_10_, PM_2.5_ and O_3_ are presented in Additional file 2: Figure S1. In this analysis, the results suggest that LA, CA had significantly higher air pollution levels than Honolulu, HI.

### Respiratory cancer cases

All respiratory cancer cases among Whites in the two selected study areas were identified from the SEER cancer registry data from 1992–2008. The SEER cancer registry, operated by the National Cancer Institute (NCI), is a system of cancer registries that includes 20 different geographic areas and covers 28% of the U.S population. Respiratory cancers were identified based on primary site using *International Classification of Diseases for Oncology,* Third Edition (ICD-O-3). Cancers being included were nose, nasal cavity and middle ear (C300-C301, C310-C319); larynx (C320-C329); lung and bronchus (C340-C349); pleura (C384); and trachea, mediastinum and other organs (C339, C381-C383, C388, C390, C398, C399). In this study, we only selected the years of 1992–2008 due to the fact that Los Angeles joined the SEER registries starting in 1992. Furthermore, since the number of Black and other minority races in Hawaii are low, and the group Asian/Pacific Islanders consists of multiple nationalities which may have significant differences in culture and dietary habits, this study only focuses on the White population.

### Outcome assessment

The causes of death were obtained from SEER, and further categorized into two groups: 1) cancer-specific death, and 2) death of other causes. Overall mortality was the main outcome of interest, and we also examined all competing risks. Survival time (unit: months) was calculated as the interval from the time of diagnosis to the time of death or to the end of the study.

### Air pollution exposure assessment

Two methods were used to assess air pollution exposure, 1) ecological exposure measurement, and 2) individual exposure measurement. In the first method, cancer cases in Honolulu County were defined as a low exposure group; and those from Los Angeles County were defined as a high exposure group because the analysis of air pollution levels between two study areas suggested that temporal variation of air pollution was very small in both study areas and differences between the two study areas were significant.

In the second method, individual exposure to air pollutants of PM_10_, PM_2.5_, and O_3_ was estimated based on date of diagnosis (year and month), survival time or follow-up time (unit: months) and county of residence. Daily air pollution monitored data in the study areas during 1992–2008 were obtained from the U.S. Environmental Protection Agency’s Air Quality System. County-level monthly means of air pollutants including PM_10_, PM_2.5_ and O_3_ were calculated using the data from all monitors within the county. For each cancer case, individual air pollution exposure during survival time or follow-up time was estimated by averaging monthly means of air pollutants during the period in the county where the subject lived. Both continuous variables and categorical variables of air pollutants were used in the following regression analyses. The categorical variables of air pollutants were categorized as high and low levels using the median concentrations of air pollutants.

### Covariates

For each cancer case, SEER contains information regarding basic demographics including age (<54, 55–69, 70–84, and 85+ years old), gender (male vs. female), marital status at diagnosis (unmarried married, separated/divorce, windowed, and unknown) and information on cancer characteristics including date of diagnosis (1992–1996, 1997–2001, 2002–2006, 2006+) site (nose, nasal cavity and middle ear, larynx, lung and bronchus, and others), and stages of cancer (In situ, localized, regional, distant, and unknown), etc. These important factors were included as confounders in the models. In addition, information on death included date of death if applicable, the cause of death, and other information.

### Statistical analyses

Descriptive statistics such as Chi-square test and t-test were applied for comparing the distributions of categorical and continuous variables among respiratory cancer cases in two study areas. Kaplain-Meier life table analyses were conducted to show survival curves between two groups and Breslow tests were performed to test the significance of the difference of survival between two groups. Cox proportional hazard models were used to assess how the influence of air pollution were related to time to overall mortality. We also estimated the model for all competing risks. The cause-specific hazard function is the fundamental concept in competing risk models, which is the hazard of death from a given cause in the presence of the competing events. Cases with a specific cause of death are compared with all those who died, but specific causes of death are not compared. Specifically, we examined deaths due to respiratory cancer and causes other than respiratory cancer. Furthermore, we examined air pollution’s effects on respiratory cancer survival among cases only from Los Angeles (over 90% of total sample were from LA) to avoid potential confounding by city-level factors (e.g., smoking rates, socioeconomic status, quality of medical care), which could differ between LA and Honolulu. Sensitivity analysis was further conducted on cases with lung and bronchus cancers only. All statistical analyses were conducted using SAS version 9.3 (Cary, NC).

## Results

From 1992–2008, there were 58,586 respiratory cancer cases among Whites including 2,393 in Honolulu County, HI and 56,193 in Los Angeles County, CA. Table 1 presents characteristics of all respiratory cancer cases among Whites by pollution level. Overall, the majority of cancer cases were lung/bronchus cancer cases (more than 90%) among Whites in both areas. The distributions of age at diagnosis, sex, marital status at diagnosis, and cancer stage at diagnosis were significantly different between two study areas.

**Table 1:** Characteristics of respiratory cancer cases among White from 1992–2008 by pollution region (n = 58,586)

**Characteristics**		**Respiratory cancer cases (%)**		P Value*
		**Honolulu**	**Los Angeles**	
		**(n = 2,393)**	**(n = 56,193)**	
Age at diagnoses	≤54	257(10.7)	5,860(10.4)	0.03
	55-69	857(35.8)	19,715(35.1)	
	70-84	1,128(47.1)	26,127(46.5)	
	85+	151(6.3)	4,489(8.0)	
Sex	Male	1,419(59.3)	30,602(54.5)	<0.01
	Female	974(40.7)	25,591(45.5)	
Marital status at diagnoses	Unmarried	273(11.4)	7,567(13.5)	<0.01
	Married	1,170(48.9)	28,622 (50.9)	
	Separated/Divorce	413(17.3)	7,137(12.7)	
	Windowed	477(19.9)	11,807(21.0)	
	Unknown	60(2.5)	1,060(1.9)	
Cancer stage	In situ	10(0.42)	280(0.50)	<0.01
	Localized	534(22.3)	10,199(18.2)	
	Regional	629(26.3)	13,049(23.2)	
	Distant	1,057(44.2)	26,037(46.3)	
	Unknown	163(6.8)	6,628(11.8)	
Primary site	Nose, nasal cavity and middle ear	29(1.2)	756(1.4)	0.55
	Larynx	169(7.1)	3,672(6.5)	
	Lung and bronchus	2,188(91.4)	51,503(91.7)	
	Others	7(0.3)	262(0.4)	
Year diagnosed	1992-1996	697(29.1)	17,977(32.0)	<0.01
	1997-2001	683(28.5)	16,724(29.8)	
	2002-2006	728(30.4)	15,490(27.6)	
	2007-2008	285(11.9)	6,002(10.7)	

*P-value from Chi-square test.

**Figure 1:**
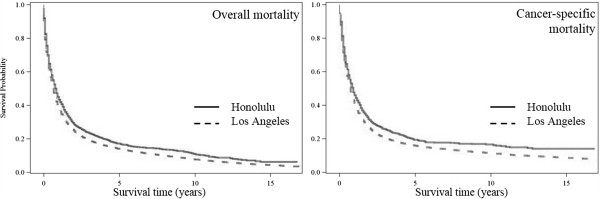
Respiratory cancer survival probability by study areas among Whites during 1992–2008 (n = 58,586).

Figure 1 shows the results of Kaplan-Meier survival analysis of all respiratory cancer cases between Honolulu, HI and Los Angeles, CA. It suggests that all respiratory cancer cases living in an area with high air pollution had a significantly higher overall and cancer-specific mortality rate than those living in an area with low air pollution(p < 0.0001) without adjusting for other covariates, while mortality due to causes other than respiratory cancer was not significant.

**Figure 2:**
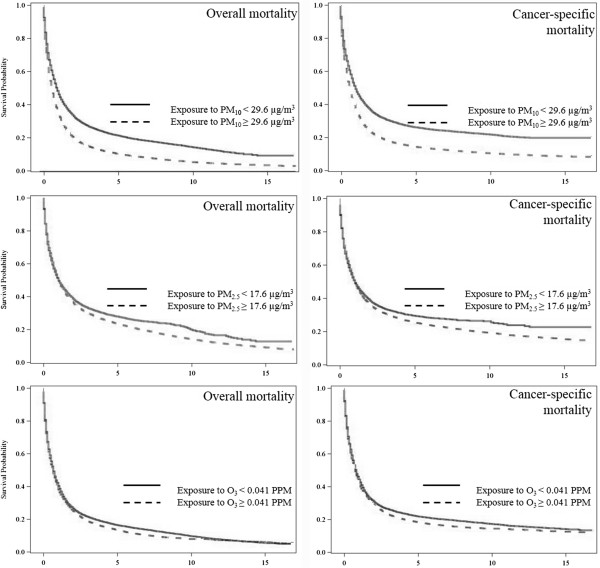
Respiratory cancer survival probability by air pollutants among Whites during 1992–2008 (n = 58,586).

Figure 2 shows the results of Kaplan-Meier survival analysis for all respiratory cancer cases survival between high and low air pollutant exposure levels. It suggests that cancer cases exposure to high levels of air pollutants including PM_10_ and PM_2.5_ had significantly low survival rate than those exposure to low levels. In addition, cases exposure to high levels of O_3_ had significantly higher overall and cancer-specific mortality rate than those with low exposure levels (p < 0.0001).

Table 2 presents the unadjusted and adjusted hazard ratios (HR) and 95% confidence intervals (CI) for death among Whites. Subjects in Los Angeles County had a statistically significant increase of 14% in overall mortality rate compared to those who lived in Honolulu County in the unadjusted model (HR: 1.14, 95% CI: 1.08-1.20). After adjustment for important factors such as age at diagnosis, marital status at diagnosis, gender, cancer stage at diagnosis, and primary sites, the rates of overall mortality from Los Angeles County remained statistically significantly higher (HR: 1.07, 95% CI: 1.02, 1.13). Consistent results were observed for respiratory cancer-specific death after adjusting for confounders (HR: 1.08, 95% CI: 1.02-1.14).

**Table 2:** Adjusted and unadjusted hazard ratios (HR) and 95% confidence intervals (CI) for respiratory cancer death from 1992–2008 among Whites (n = 58,586)

**Characteristics**		**Overall death**		**Cancer-specific death**	
		**Unadjusted HR**	**Adjusted HR**^**a**^	**Unadjusted HR**	**Adjusted HR**^**a**^
		**(95% CI)**	**(95% CI)**	**(95% CI)**	**(95% CI)**
Air pollution level ^b^	Low	1.00	1.00	1.00	1.00
	High	1.14(1.08–1.20) *	1.07(1.02–1.13) *	1.15(1.09–1.22) *	1.08(1.02–1.14) *
Age at diagnoses	≤54	1.00	1.00	1.00	1.00
	55–69	1.29(1.25–1.34) *	1.30(1.25–1.35) *	1.21(1.16–1.25) *	1.21(1.16–1.26) *
	70–84	1.72(1.66–1.78) *	1.73(1.67–1.80) *	1.49(1.43–1.54) *	1.49(1.44–1.55) *
	85+	2.70(2.58–2.83) *	2.57(2.43–2.71) *	2.25(2.14–2.36) *	2.12(2.00–2.25) *
Marital status	Unmarried	1.00	1.00	1.00	1.00
	Married	0.89(0.86–0.91) *	0.82(0.79–0.85) *	0.91(0.88–0.94) *	0.86(0.83–0.89) *
	Separated/Divorce	1.01(0.97–1.05)	0.97(0.93–1.01)	1.03(0.99–1.07)	0.99(0.95–1.04)
	Widowed	1.26(1.22–1.30) *	1.02(0.98–1.06)	1.23(1.19–1.28) *	1.06(1.01–1.10) *
Sex	Male	1.00	1.00	1.00	1.00
	Female	0.97(0.95–0.99) *	0.82(0.81–0.84) *	0.99(0.97–1.01)	0.84(0.82–0.86) *
Cancer stage	In situ or Localized	1.00	1.00	1.00	1.00
	Regional	1.98(1.91–2.06) *	1.96(1.89–2.03) *	2.54(2.43–2.65) *	2.46(2.36–2.57) *
	Distant	5.26(5.09–5.44) *	5.04(4.87–5.21) *	7.20(6.91–7.49) *	6.64(6.37–6.92) *
Primary site	Lung and bronchus	1.00	1.00	1.00	1.00
	Nose, nasal cavity and middle ear	0.38(0.34–0.42) *	0.49(0.44–0.55) *	0.32(0.29–0.36) *	0.43(0.38–0.49) *
	Larynx	0.32(0.31–0.34) *	0.54(0.51–0.57) *	0.23(0.21–0.24) *	0.41(0.38–0.44) *
	Others	0.36(0.30–0.42) *	0.38(0.30–0.48) *	0.38(0.31–0.46) *	0.40(0.31–0.52) *
Year diagnosed	2007–2008	1.00	1.00	1.00	1.00
	2002–2006	1.04(1.00–1.09)	1.25(1.20–1.32) *	1.06(1.01–1.11) *	1.09(1.04–1.15) *
	1997–2001	1.04(0.99–1.08)	1.18(1.12–1.24) *	1.04(0.99–1.09)	1.20(1.14–1.26) *
	1992–1996	1.06(1.02–1.11) *	1.25(1.20–1.32) *	1.05(1.00–1.10)	1.27(1.21–1.34) *

*Statistically significant at alpha = 0.05.

^a^ Adjusted hazard ratios are adjusted for Age at Diagnoses, Sex, Marital status at diagnoses, Cancer stage, Primary site, and Year diagnosed.

^b^ Subjects from Los Angeles, CA were defined as high air pollution exposure group while those from Honolulu, HI were defined as low air pollution exposure group.

Table 3 presents the unadjusted and adjusted hazard ratios (HR) and 95% confidence intervals (CI) of death due to each air pollutants among Whites. The results suggest that exposure to air pollutants including PM_10_, PM_2.5,_ and O_3_ was significantly associated with both overall mortality and cause-specific mortality for respiratory cancer patients. Consistent results were observed when only cases in Los Angeles were included. Furthermore, similar results were found in the sensitivity analysis when only cases with lung and bronchus cancer were included (results not shown).

**Table 3:** Hazard ratios (HR) of death by air pollutants for respiratory cancer cases among Whites from 1992-2008

**Air pollutant**^**a**^		**Overall death**		**Cancer-specific death**	
		**Unadjusted HR**	**Adjusted HR**^**b**^	**Unadjusted HR**	**Adjusted HR**^**b**^
		**(95% CI)**	**(95% CI)**	**(95% CI)**	**(95% CI)**
Honolulu and Los Angeles(n = 58,586)					
PM_10_	Low (< 29.6 μg/m^3^)	1.00	1.00	1.00	1.00
	High (≥ 29.6 μg/m^3^)	1.41(1.39–1.44) ^*^	1.63(1.58–1.67) ^*^	1.39(1.36–1.42) ^*^	1.56(1.51–1.61) ^*^
	Continuous variable (per 10 μg/m^3^)	1.40(1.37–1.42) ^*^	1.48(1.44–1.52) ^*^	1.37(1.35–1.40) ^*^	1.43(1.39–1.46) ^*^
PM_2.5_	Low (< 17.6 μg/m^3^)	1.00	1.00	1.00	1.00
	High (≥ 17.6 μg/m^3^)	1.08(1.05–1.11) ^*^	2.04(1.96–1.12) ^*^	1.07(1.04–1.10) ^*^	1.97(1.89–2.05) ^*^
	Continuous variable (per 5 μg/m^3^)	1.23(1.21–1.25) ^*^	1.57(1.53–1.61) ^*^	1.20(1.18–1.22) ^*^	1.49(1.45–1.53) ^*^
O_3_	Low (< 0.041 PPM)	1.00	1.00	1.00	1.00
	High(≥ 0.041 PPM)	1.07(1.05–1.09) ^*^	1.04(1.01–1.06) ^*^	1.07(1.05–1.10) ^*^	1.04(1.02–1.07) ^*^
	Continuous variable (per 10 PPB)	1.08(1.06–1.09) ^*^	1.04(1.03–1.06) ^*^	1.09(1.07–1.11) ^*^	1.06(1.04–1.07) ^*^
Los Angeles (n = 56,193)					
PM_10_	Low (< 29.7 μg/m^3^)	1.00	1.00	1.00	1.00
	High (≥ 29.7 μg/m^3^)	1.46(1.43–1.49) ^*^	1.77(1.72–1.83) ^*^	1.44(1.41–1.48) ^*^	1.70(1.64–1.76) ^*^
	Continuous variable (per 10 μg/m^3^)	1.51(1.48–1.54) ^*^	1.91(1.85–1.98) ^*^	1.47(1.44–1.50) ^*^	1.79(1.73–1.86) ^*^
PM_2.5_	Low (< 17.9 μg/m^3^)	1.00	1.00	1.00	1.00
	High (≥ 17.9 μg/m^3^)	1.09(1.06–1.12) ^*^	2.24(2.15–2.33) ^*^	1.07(1.04–1.10) ^*^	2.13(2.04–2.22) ^*^
	Continuous variable (per 5 μg/m^3^)	1.42(1.39–1.45) ^*^	2.51(2.43–2.59) ^*^	1.35(1.32–1.39) ^*^	2.28(2.20–2.36) ^*^
O_3_	Low (< 0.041 PPM)	1.00	1.00	1.00	1.00
	High(≥ 0.041 PPM)	1.15(1.13–1.17) ^*^	1.12(1.09–1.15) ^*^	1.15(1.13–1.18) ^*^	1.12(1.09–1.15) ^*^
	Continuous variable (per 10 PPB)	1.07(1.05–1.09) ^*^	1.04(1.02–1.06) ^*^	1.09(1.07–1.11) ^*^	1.06(1.04–1.08) ^*^

* Statistically significant at alpha = 0.05.

^a^ High and low levels were categorized using the median concentration of air pollutant.

^b^ Adjusted hazard ratios are adjusted for Age at Diagnoses, Sex, Marital status at diagnoses, Cancer stage, Primary site, and Year diagnosed.

## Discussion

Our study suggests that among diagnosed respiratory cancer cases, those living in a heavily-polluted area had significantly shorter survival times compared to those living in a less-polluted area. Further, based on the analysis of individual exposure assignment, we also found that exposure to air pollutants including PM_10_, PM_2.5_ and O_3_ had adverse effects on the length of respiratory cancer survival. To our knowledge, this study is the first and largest population-based study to investigate long-term effects of air pollution on respiratory cancer survival. Our study raised concerns that air pollution exposure may have a great impact on the length of survival for respiratory cancer patients. More importantly, this study also brings attention to the need for preventive efforts to protect cancer patients from air pollution, which is currently overlooked in cancer patient care.

The effect of air pollution on the length of survival of respiratory cancer patients is biologically plausible. Long-term exposure to air pollution has been extensively linked with mortality and cancer-specific mortality [19-23]. Previous studies also suggests that air pollution, especially PM_2.5_ and O_3_, has been associated with early mortality in susceptible populations with chronic conditions such as COPD, diabetes, heart failure, or myocardial infarction [24,25]. These results are consistent with the findings from this study. The biological mechanisms by which air pollution can impact the length of survival of respiratory cancer patients is still unclear. However, it is clear that the respiratory system is an organ that is most directly affected by air pollution which carries many types of toxic chemicals including those with carcinogenic potential [26]. These pollutants could reach the wall of respiratory system and even into the blood and other organs, and induce systematic inflammation [27]. Respiratory cancer patients are at increased risk for impaired respiratory function because of cancer. Furthermore, side effects such as depressed immunity and decreased resistance to infection that accompanies cancer therapy make this group even more susceptible to contaminants in the ambient air. Thus, exposure to air pollution could further reduce their respiratory function, cause respiratory problems and other complications and/or make them vulnerable to other risk factors [28]. Consequently, the adverse effect of air pollution could significantly decrease survival time for cancer patients and cause an earlier death.

In this study, we selected two study areas with documented significantly different air pollution levels. In Los Angeles, CA, the average annual PM_2.5_ was 18.1 μg/m^3^ during 1999–2008, which is well above the U.S. EPA’s annual PM_2.5_ standard of 15 μg/m^3^. Meanwhile, the average annual PM_2.5_ during 1999–2008 in Honolulu, HI was only 4.3 μg/m^3^, which is much below the annual standard (Please refer to Additional file 1: Table S1). Over the study period, air pollution level has decreased in the LA but little temporal variation was observed in Honolulu. Overall, the difference of air quality between two places remained consistently significant during 1992–2008 (Please refer to Additional file 2: Figure S1). We selected LA as a highly-polluted area and Honolulu as a low-polluted area in this study and this ecological exposure assignment might reduce misclassification of air pollution exposure because of a significant difference and low temporal variation of air pollution levels between two areas, i.e. variations of air pollution between counties (LA vs. Honolulu) were much greater than variations within a county. In addition, cancer patients were unlikely to move, which would also minimize the potential errors in exposure assessment when we used this ecological exposure assignment. However, we cannot rule out the possibility that the observed differences in cancer survival time between two locations could be also due to other factors that differ across the two locations, such as smoking rate, socioeconomic status and quality of medical care etc. Furthermore, individual measurements of air pollution were also estimated using the county-level air pollution data from the U.S. EPA air monitoring system. The results from the analyses of individual exposure assignment also suggest that exposure to higher air pollution may have adverse effects on the length of survival from respiratory cancers in this study. However, since Honolulu has much lower pollution levels than LA, most of the individuals with high pollution exposure would be from LA and most with low pollution exposure will be from Honolulu. As a result, any observed association between pollution and cancer survival could still be attributed to other factors that differ between the two cities. For example, solar ultraviolet-B (UVB) and vitamin D were linked to cancer mortality in many studies [29-33]. Solar UVB doses are much higher in Honolulu than in LA. These unselected factors may explain the observed associations. To further eliminate this possibility, we performed a sensitive analysis to investigate the association between air pollution and cancer survival in each city. As LA had over 95% of cases and higher air pollution level, we limited our analysis in LA. The results from this analysis remain consistent. Therefore, the observed association between air pollution and respiratory cancer survival in this study is unlikely explained by potential confounding due to city-level factors. Finally, although individual demographical factors and stage of cancer were adjusted for in this study, other unselected factors such as dietary, medical care, and lifestyle factors of smoking and alcohol consumption may also confound the observed association. As information on these factors is not available in this study, we could not control for their potential confounding effects. However, although demographic distributions between LA and Honolulu are much different, we only studied one race of White in this study. Thus, the influences of different culture and dietary habits are unlikely to explain the significant differences in length of survival from respiratory cancer. Moreover, both areas in this study are urban areas with the same healthcare system in the United States, which is also improbable to account for this significant difference. In addition, as cancer patients, current tobacco use and alcohol consumption are not common; thus the confounding effects of these factors should be limited [34]. Further, the poverty rates among Whites in the two study areas, according to the U.S census data, are very close, i.e. 10% in Honolulu, HI and 11% in Los Angeles, CA [35]. Therefore, our preliminary data are promising and convincing, suggesting that this field is worthy of further investigation.

## Conclusion

In summary, this study revealed that air pollution exposure may have deleterious effects on length of survival from respiratory cancer patients. With 12.5 million people living with cancer in the U.S. and an annual incidence and mortality rates of approximately 500 and 200 per 100,000, respectively, it is important to pay attention to potential effects of air pollution on cancer survival. Careful assessment of the potential deleterious effects of air pollution among this susceptible group is also necessary for improvement of cancer survival and establishment of sound regulatory policy to promote the public health and welfare. As several limitations exist in this study, additional research is clearly warranted.

### What this paper adds

Air pollution is a global environmental issue and, to our knowledge, no study has been conducted to investigate health effect of air pollution on length of survival among cancer patients. In addition, protection of this susceptible group of cancer patients from environmental threats has largely been overlooked in standard clinical care. In this study, we selected two study areas with significantly different air pollution levels: Honolulu, HI (low levels of air pollution) vs. Los Angeles, CA (high levels of air pollution), and then estimated individual air pollution exposure during survival period or study period using air monitor data and further investigated if exposures to particular matter and ozone have significant impacts on the length of cancer survival. Our study suggests that exposure to high levels of air pollution had adverse effects on length of survival compared with low levels of air pollution after adjusting for important confounders such age, gender, race, diagnosis stage of cancer and primary sites. This study also calls for attention to protections from environmental contaminants for this susceptible group.

## Competing interests

The authors declare that they have no competing interests.

## Authors’ contributions

XX conceived of the study, and participated in its design and coordination and draft the manuscript. SH participated in the design of the study, performed the statistical analysis, and drafted the manuscript. HK was involved in design of the study, results interpretation, manuscript drafting. HH performed the statistical analysis, and helped revise the manuscript. BC helped interpret results and draft the manuscript. CL helped analyze the data and drafted the manuscript. All authors read and approved the final manuscript.

## Pre-publication history

The pre-publication history for this paper can be accessed here:

http://www.biomedcentral.com/1471-2458/13/800/prepub

## Supplementary Material

Additional file 1: Table S1. Means of annual averages of PM_10_, PM_2.5_ and O_3_ between 1992–2008 in Los Angeles, CA and Honolulu, HI. Click here for file

Additional file 2: Figure S1. Annual average concentrations of air pollutants between Los Angeles, CA and Honolulu, HI in 1992–2008. Click here for file
