# Supplementary material for: Health effects of air pollution on length of respiratory cancer survival
Source: BMC Public Health. 2013 Sep 3;13:800. doi: 10.1186/1471-2458-13-800 (PMC3766670; doi:10.1186/1471-2458-13-800)
Supplement: Additional file 1: Table S1. — Means of annual averages of PM10, PM2.5 and O3 between 1992–2008 in Los Angeles, CA and Honolulu, HI. [file 1471-2458-13-800-S1.doc]

Additional file 1: Means of annual averages of PM_10_, PM_2.5_ and O_3_ between 1992-2008 in Los Angeles, CA and Honolulu, HI.

| Air pollutants | Year monitored | Study Locations | | P-value* |
| --- | --- | --- | --- | --- |
|  |  | Los Angeles, CA | Honolulu, HI |  |
| PM_10_ (μg/m^3^) | 16 | 33.7±4.4 | 15.0±1.0 | <0.0001 |
| PM_2.5_ (μg/m^3^) | 9 | 18.1±2.7 | 4.3±0.2 | <0.0001 |
| Ozone (ppm) | 16 | 0.042±0.0035 | 0.024±0.0040 | <0.0001 |
| *P-value from the two-sample t-test | | | | |
