# Supplementary figures and images for: Health effects of air pollution on length of respiratory cancer survival
Source: BMC Public Health. 2013 Sep 3;13:800. doi: 10.1186/1471-2458-13-800 (PMC3766670; doi:10.1186/1471-2458-13-800)

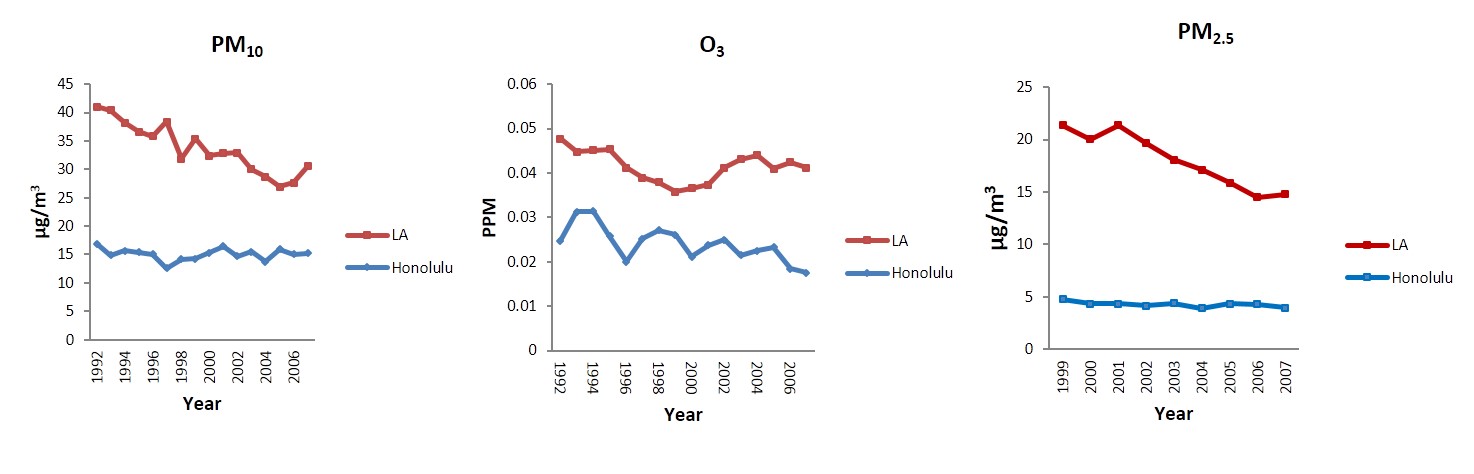

Supplement: Additional file 2: Figure S1. — Annual average concentrations of air pollutants between Los Angeles, CA and Honolulu, HI in 1992–2008. [file 1471-2458-13-800-S2.jpeg]
